# Differential proteomics of placentas reveals metabolic disturbance and oxidative damage participate yak spontaneous miscarriage during late pregnancy

**DOI:** 10.1186/s12917-022-03354-w

**Published:** 2022-06-27

**Authors:** Jie Pei, Shoubao Zhao, Mancai Yin, Fude Wu, Jiye Li, Guomo Zhang, Xiaoyun Wu, Pengjia Bao, Lin Xiong, Weiru Song, Yang Ba, Ping Yan, Rende Song, Xian Guo

**Affiliations:** 1 Key Laboratory of Yak Breeding Engineering of Gansu Province, Lanzhou Institute of Husbandry and Pharmaceutical Sciences, Chinese Academy of Agricultural Sciences, Lanzhou, China; 2 Key Laboratory of Animal Genetics and Breeding On Tibetan Plateau, Ministry of Agriculture and Rural Affairs, Lanzhou, China; 3 Yak Breeding and Extending Service Centre in Qinghai Province, Datong, China; 4 Animal Disease Prevention and Control Centre of Yushu Tibetan Autonomous Prefecture, Yushu, China

**Keywords:** Yak, Abortion, Placenta, iTRAQ, Proteomics, Metabolism, Oxidative stress

## Abstract

**Background:**

High spontaneous miscarriage rate in yak, especially during late pregnancy, have caused a great economic loss to herdsmen living in the Qinghai-Tibet plateau. However, the mechanism underlying spontaneous miscarriage is still poorly understood. In the present study, placenta protein markers were identified to elucidate the pathological reasons for yak spontaneous miscarriage through isobaric tags for relative and absolute quantification (iTRAQ) proteomic technology and bioinformatic approaches.

**Results:**

Subsequently, a total of 415 differentially expressed proteins (DEPs) were identified between aborted and normal placentas. The up-regulated DEPs in the aborted placentas were significantly associated with “spinocerebellar ataxia”, “sphingolipid signalling”, “relaxin signalling”, “protein export”, “protein digestion and absorption” and “aldosterone synthesis and secretion” pathway. While the down-regulated DEPs in the aborted placentas mainly participated in “valine, leucine and isoleucine degradation”, “PPAR signalling”, “peroxisome”, “oxidative phosphorylation”, “galactose metabolism”, “fatty acid degradation”, “cysteine and methionine metabolism” and “citrate cycle” pathway.

**Conclusions:**

The results implied that the identified DEPs could be considered as placental protein markers for yak miscarriage during late pregnancy, and biomacromolecule metabolic abnormality and oxidative damage might be responsible for the high spontaneous miscarriage rate in yak. These findings provide an important theoretical basis for deciphering the pathologic mechanism of late spontaneous miscarriage in yak.

**Supplementary Information:**

The online version contains supplementary material available at 10.1186/s12917-022-03354-w.

## Background

Yak (*Bos grunniens*) is an important economic source for herdsmen living in the Qinghai-Tibet plateau because of its use as a source of meat, milk, leather and wool [1]. The greater number of yaks means the higher economic income for herdsmen [2]. However, low reproduction performance, attributable in part to high miscarriage rate, is the main constraint to increasing yak population [3]. The miscarriage rates of yaks in Guoluo Tibetan Autonomous County and Haiyan Tibetan Autonomous County (Qinhai province, Northwest of China) are highest in the Qinghai-Tibet plateau, even reaching over 30% in some years, thus causing massive economic losses to the local yak industry. Consequently, elucidating the mechanism of miscarriage pathogenesis is of particular importance for reversing the current situation of yak miscarriage.

In mammals, establishment and maintenance of pregnancy require an appropriate reciprocal interaction between matrix and its conceptus and the interaction is regulated by actions of maternal secretions and conceptus hormones [4, 5]. During pregnancy, endometrial gland morphogenesis promotes endometrium to enhance expression of secretory proteins that are transported to foetus by areolae in human placenta [6]. A uterine gland knockout ewe model leading to early pregnancy loss demonstrates that the endometrial epithelial secretions are vital to peri-implantation blastocyst survival and development [4]. On the other hand, hormone signals sent by conceptus to maternal system can promote maternal pregnancy recognition to keep conceptus continuously developing in cattle [7]. The conceptus hormones act on the uterus in a paracrine manner to establish and maintain pregnancy. In most mammals, hormones from conceptus trophoblast keep progesterone production, and further maintain functional corpus luteum by acting on endometrium to prevent uterine release of luteolytic prostaglandin F2α [6].

An accurate differential diagnosis is a challenge for miscarriage. The reasons of miscarriage can be mainly classified into infectious and non-infectious etiology [8, 9], the former induced by bacteria, chlamydia and viruses [9], and the latter caused by genetic and non-genetic disorders [10, 11]. In regard to bacteria, seropositivity to brucella infection is implicated in yak miscarriage, resulting in the decline of yak populations in the mountain regions of Nepal [12]. Yak herds with miscarriages occurrence within a previous year are 2.3 times more likely to be seropositive for brucella than herds without miscarriages [12]. *Chlamydia abortus* (*C. abortus*), a family of Gram-negative obligate intracellular bacteria, is another major abortigenic agent for yak [13–15]. The chlamydia targets yak placentas where it causes pathological damage affecting pregnancy outcome in the final two to three weeks of gestation [15]. Up to now, three types of viruses, herpesvirus-1, Akabane virus and bovine viral diarrhea virus [16], leading to miscarriage are identified in yak [17, 18]. Serological diagnosis of bovine herpesvirus-1 reveals 60.1% of overall seroprevalence in a yak farm with miscarriage history [17]. Infections caused by Akabane virus and bovine viral diarrhea virus also bring about miscarriage, stillbirth and malformation in yak [18, 19]. However, the non-infectious elements of spontaneous yak miscarriage, such as genetic and immune status, remain to be illuminated.

Molecular and cellular communication between bovine matrix and foetus is established by bidirectional transplacental signal exchange [20]. So, discovering placenta proteins causing miscarriage is extremely necessary to target reliable biomarkers for clinical diagnosis prior to miscarriage, and to provide new strategies for improving pregnancy outcome and enhancing reproductive efficiency in yak. In the present study, comparative isobaric tags for relative and absolute quantification (iTRAQ) proteomics was applied to identify protein markers related to miscarriage in yak placenta. Furthermore, bioinformatic methods were used to reveal the pathogenesis underlying spontaneous miscarriage in late yak pregnancy, based on the differential proteomics results. The findings are beneficial to facilitate development of suitable therapeutic management for yak miscarriage.

## Results

### Total protein identification in the yak placentas

**Fig. 1:**
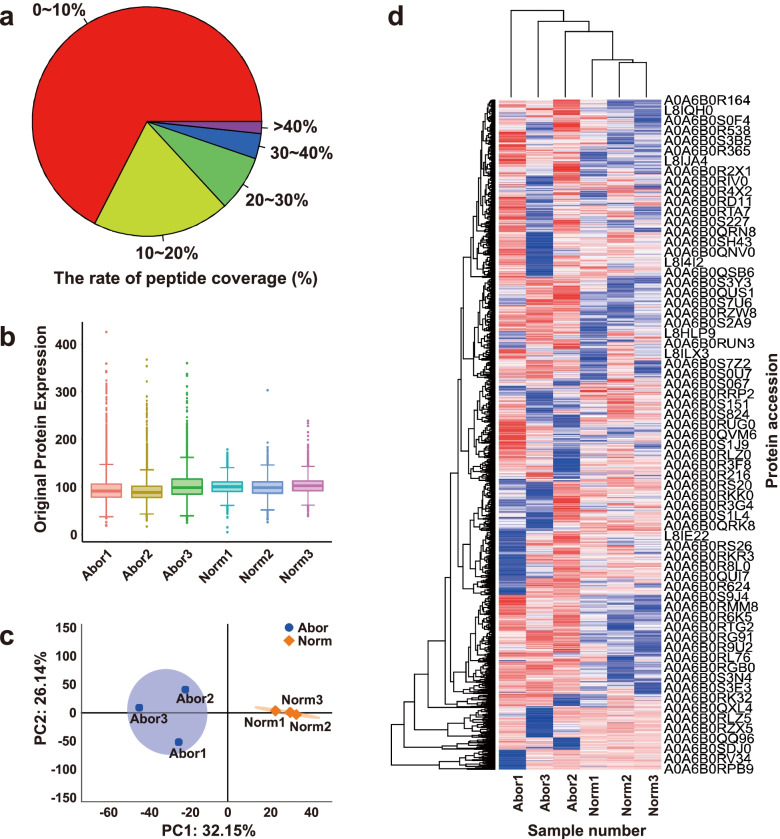
Expression status, principal component analysis (PCA) and heatmap of the total identified proteins in the yak placentas. (**a**) Pie chart shows the rate of identified peptide coverage; (**b**) Boxplot indicates the total protein expression in the different placenta samples; (**c**) PCA biplot exhibits the aggregation of the intragroup samples based on the first two principal components; (**d**) Heatmap demonstrates the global expression difference of the total proteins between the normal and aborted placenta samples

The yak placenta samples were subjected to iTRAQ analysis to find differentially expressed proteins (DEPs) between normal and aborted placentas. The mass spectrometry proteomics data have been deposited to the ProteomeXchange Consortium (http://proteomecentral.proteomexchange.org) via the iProX partner repository [21] with the dataset identifier PXD025428. A total of 16,752 unique peptides were identified with 0.01 false discovery rate (FDR) threshold (Additional file 1). The rate of peptide coverage is presented as a pie chart (Fig. 1a). The unique peptides were mapped to the yak genomic reference database, while 3,738 proteins were identified in all the placenta sample (Additional file 2). Subsequently, 2,850 trusted proteins were screened out from the total identified proteins (Additional file 3).

### Expression pattern analyses of the total proteins from the yak placentas

The box plot of protein expressions shows the lower variability of intragroup than that of intergroup, demonstrating the similar protein expression patterns in the same groups (Fig. 1b). The distribution of protein expression levels in the abortive group is wider than that in the normal group (Fig. 1b), implying there are many abnormally expressed proteins in the aborted placentas. The placenta samples are clustered into two groups in the biplot graph based on principal component analysis (PCA) (Fig. 1c), exactly as they were classified into abortive group and normal group, indicating the compositional similarities/differences among the placenta samples. Through constructing the heatmap of the total identified proteins (Fig. 1d), the yak placenta samples are also gathered into two groups as grouped by the placenta statuses, indicating the two highly clustered sampled-based within-group expression patterns.

### Screening and expression pattern analyses of the differentially expressed proteins

**Fig. 2:**
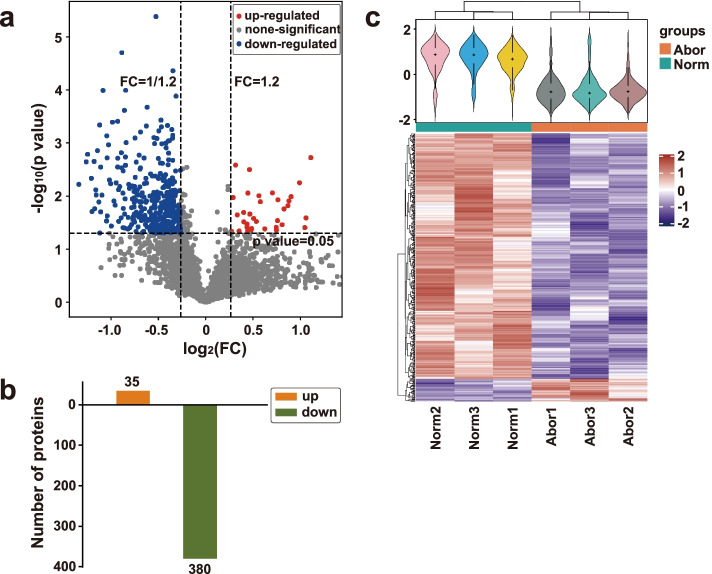
Expression pattern of the differentially expressed proteins (DEPs) between the normal and abortive group. (**a**) Volcano plot illustrates the DEPs between the normal and abortive group; (**b**) Bar plot exhibits the numbers of the up- and down-regulated DEPs; (**c**) Heatmap demonstrates the DEP expression difference between the normal and aborted placenta samples

Each trusted protein with a fold change greater than 1.2 and a *p*-value less than 0.05 was determined as a DEP in the Student’s t-test. A total of 415 DEPs, including 35 up-regulated and 380 down-regulated DEPs (Additional files 4 and 5), were identified in the abortive group compared with the normal group (Fig. 2b). The volcano plot for the total proteins was drawn to demonstrate the up- and down-regulated DEPs in the abortive group compared with the normal group. In the volcano plot, there are more down-regulated DEPs than up-regulated ones in the abortive group (Fig. 2a). The differential expression profiling between the two placenta groups is visualized in the heatmap according to the relatively expressed levels of the DEPs (Fig. 2c).

### Gene ontology enrichment for the differentially expressed proteins

**Fig. 3:**
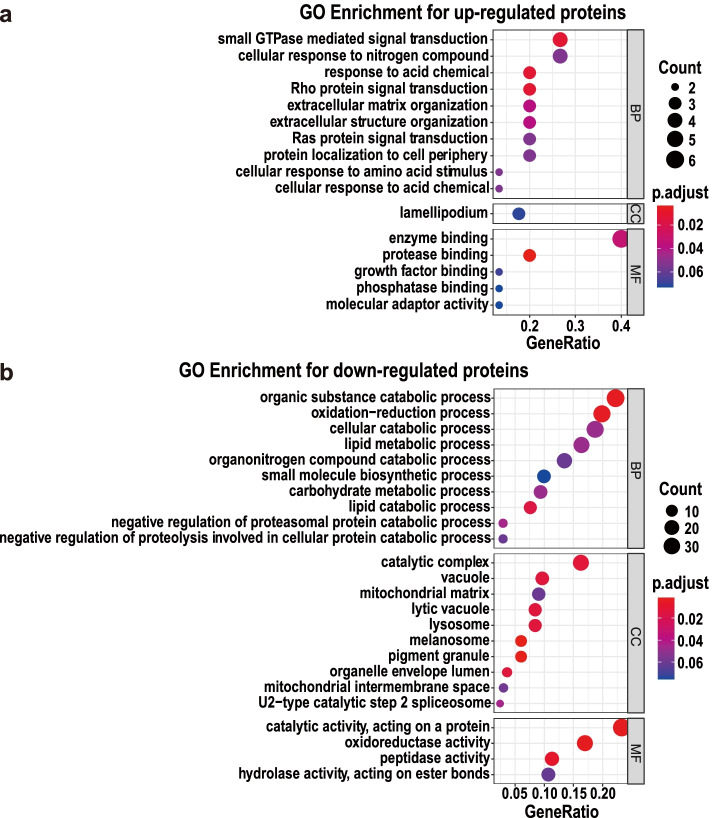
Bubble diagrams of the gene ontology (GO) enrichment analysis for the differentially expressed proteins (DEPs) between the normal and abortive group. (**a**) Bubble diagram demonstrates that the up-regulated DEPs in the abortive group participate signal transduction, cellular response, extracellular structure organization and protein localization; (**b**) Bubble diagram manifests that the down-regulated DEPs in the abortive group take parts in lipid, protein and carbohydrate metabolic process and redox balance regulation

The Gene ontology (GO) analysis was performed for the up- and down-regulated DEPs separately. The terms of the GO functional enrichment analysis were classified into three categories, namely biological process (BP), cellular component (CC) and molecular function (MF) (Fig. 3). GO terms with adjusted *p*-value greater than 0.05 were considered as significant difference in the enrichment targets (Additional files 6 and 7). The top 10 enriched terms of each category for the up-regulated DEPs in the abortive group are exhibited as the upper bubble plot in Fig. 3a. The functional enrichment of the BP category for the up-regulated proteins shows that the enriched proteins mainly participate in “response to acid chemical”, “Rho protein signal transduction”, “small GTPase mediated signal transduction”, “extracellular matrix organization”, “extracellular structure organization”, “cellular response to nitrogen compound”, “Ras protein signal transduction”, “cellular response to amino acid stimulus”, “protein localization to cell periphery” and “cellular response to acid chemical” (Additional file 6). Most of the top terms contained COL1A1, COL1A2 and FLOT1, indicating the three proteins playing crucial roles in these BP terms (Additional file 4). The GO enrichment of the up-regulated DEPs suggests that signal transduction, cellular response, extracellular structure organization and protein localization were disturbed primarily in the aborted yak placentas.

The top 10 enriched terms of the BP category for the down-regulated DEPs in the abortive group are shown in the lower bubble plot of Fig. 3b. The BP category enrichment reveals that the down-regulated DEPs play important roles in “organic substance catabolic process”, “oxidation–reduction process”, “lipid catabolic process”, “negative regulation of proteasomal protein catabolic process”, “lipid metabolic process”, “cellular catabolic process”, “carbohydrate metabolic process”, “negative regulation of proteolysis involved in cellular protein catabolic process”, “organonitrogen compound catabolic process” and “small molecule biosynthetic process” (Additional file 7). With respect to the protein functions in the GO enrichment, the results indicate that GCDH, ACADM, ASAH1, SCARB1, PLA2G15, ETFB, ACOX1 and NAGA take parts in lipid metabolic process; GABARAPL2, PARK7, HSP90AB1, ALAD and SGTA participate protein metabolic process; ACADM, FABP5, GAPDH and FBP1 involve in carbohydrate metabolic process; and TXN2, TXNRD2, CAT, LHPP, PPA2, SORD, SOD1, ALDH7A1 and PRDX2 are in connection with process oxidation–reduction (Additional file 5). The GO enrichment for the down-regulated DEPs implies that protein, carbohydrate and lipid metabolic process and redox balance regulation were abnormal in the aborted yak placentas.

### Kyoto encyclopaedia of genes and genomes enrichment for the differentially expressed proteins

The Kyoto encyclopaedia of genes and genomes (KEGG) pathway enrichment was also conducted to interpret gene sets of interest for the up- and down-regulated DEPs separately. The pathways with adjusted *p*-value greater than 0.05 were considered as significantly enriched targets (Additional files 8 and 9).

**Fig. 4:**
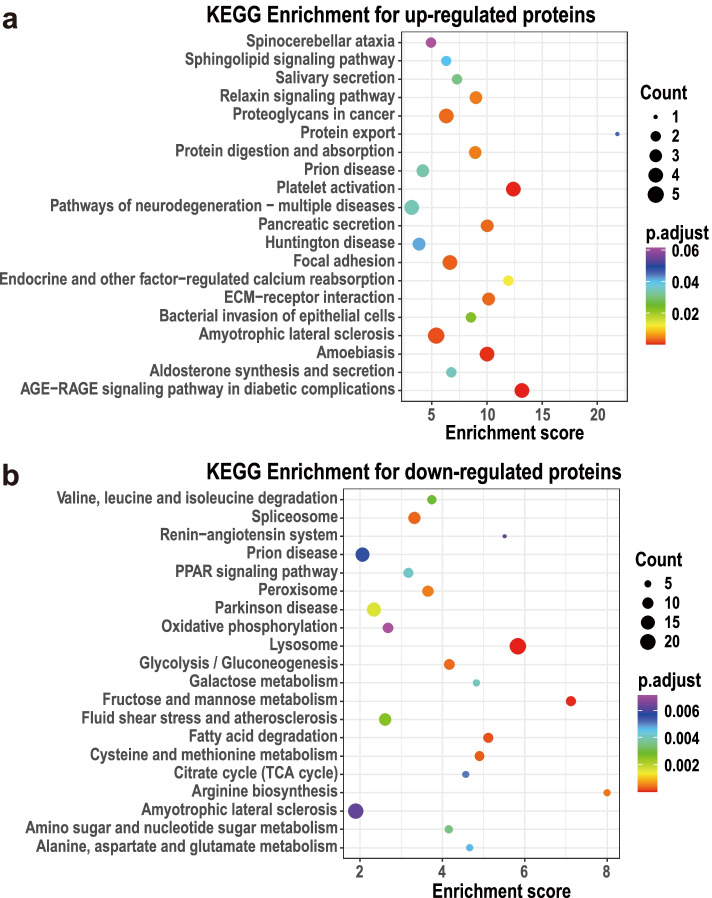
Bubble diagrams of Kyoto encyclopaedia of genes and genomes (KEGG) pathway enrichment for the differentially expressed proteins (DEPs) between the normal and abortive group. (**a**) Bubble diagram demonstrates that the up-regulated DEPs participate in relaxin signal, protein metabolism and defend against pathogens; (**b**) Bubble diagram indicates that the down-regulated DEPs take parts in amino acid degradation, glycometabolism, lipid metabolism and redox balance

The 20 top KEGG pathways for the up-regulated DEPs are shown in the upper bubble plot (Fig. 4a), primarily including “sphingolipid signalling pathway”, “relaxin signalling pathway”, “protein export”, “protein digestion and absorption”, “bacterial invasion of epithelial cells”, “amoebiasis”, “aldosterone synthesis and secretion” and “AGE-RAGE signalling pathway in diabetic complications”. In line with the enriched BP terms, the most of KEGG pathways also contain COL1A1, COL1A2 and FLOT1 (Additional file 8). The KEGG enrichment for the up-regulated DEPs suggests that lipid metabolism, protein catabolism, saccharide metabolism and relaxin signalling operated abnormally in the aborted placentas.

The 20 top KEGG pathways for the down-regulated DEPs are exhibited in the lower bubble plot (Fig. 4b), mainly containing “Valine, leucine and isoleucine degradation”, “PPAR signalling pathway”, “peroxisome”, “oxidative phosphorylation”, “glycolysis/gluconeogenesis”, “galactose metabolism”, “fructose and mannose metabolism”, “fatty acid degradation”, “cysteine and methionine metabolism”, “citrate cycle”, “arginine biosynthesis”, “amino sugar and nucleotide sugar metabolism” and “alanine, aspartate and glutamate metabolism”. The detailed information of the KEGG pathways are stored in Additional file 9. As for the protein functions in the KEGG pathways, the findings indicate that ACSL5, ACSL6, NPC1, NPC2, HADH, ALDH7A1, GCDH, ACADM and CPT2 take parts in the pathways involved in lipid metabolic process; GOT1, GOT2, BCAT1, ASS1, ALDH7A1, HADH, HIBADH, ACADM and PCCB participate the pathways related to protein metabolic process; SORD, HKDC1, PGM2, DMM2, AKR1B1, IDH1, PC, DLAT, FH, FBP1, ALDH7A1, GAPDH, CAT and PCCB involve in carbohydrate metabolic process; and CAT, IDH, ACSL6, ACSL5, CPT2, ACADM, ATP6V1A, ATP6V0C, PPA2 and LHPP are in connection with oxidation–reduction pathway (Additional file 9). The KEGG enrichment for the down-regulated DEPs suggest that amino acid degradation, glycometabolism, lipid metabolism and redox balance were disturbed principally in the aborted placentas.

### Protein to protein interaction among the differential expressed proteins

**Fig. 5:**
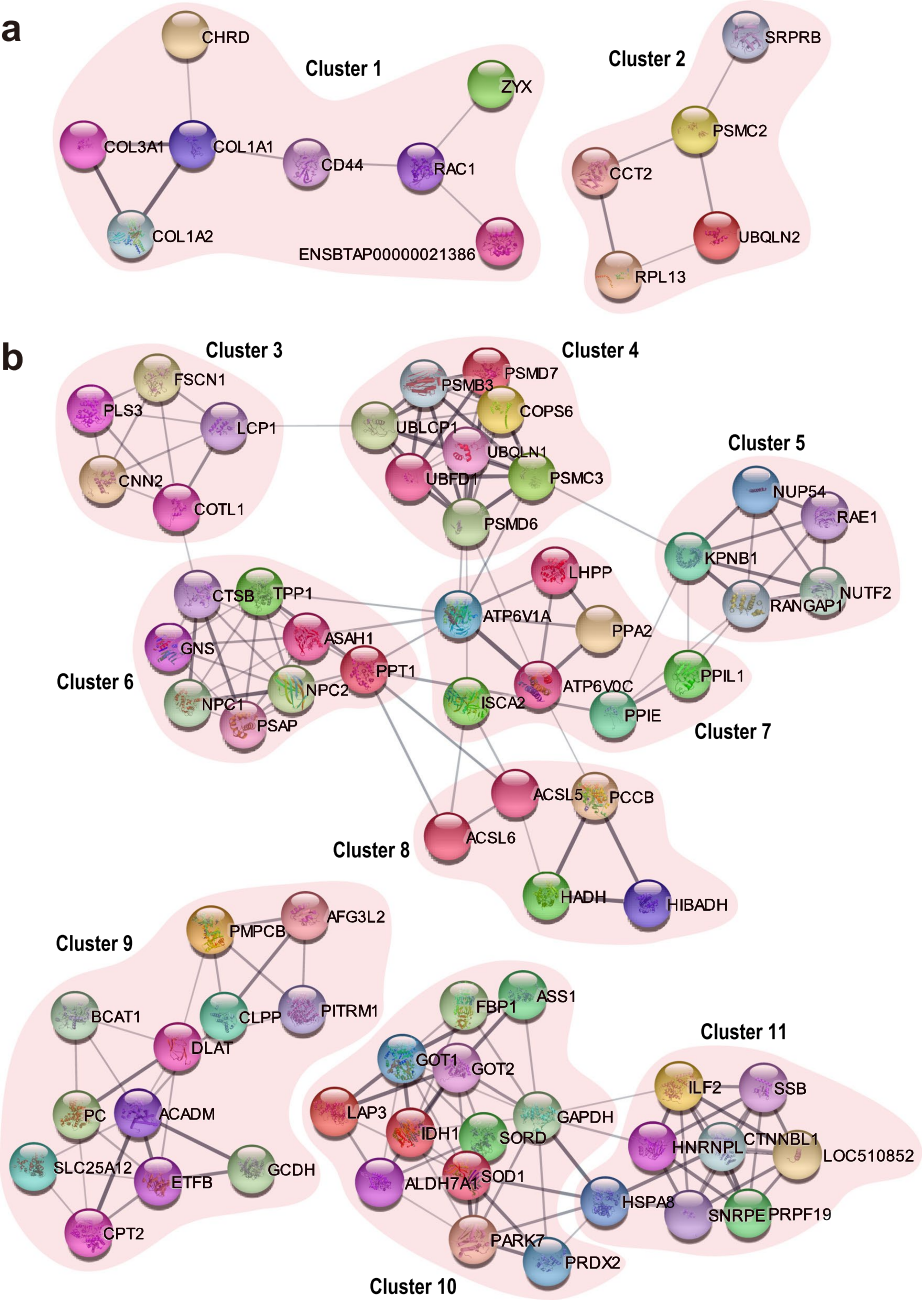
Networks of protein to protein interaction (PPI) of the differentially expressed proteins (DEPs) screened by molecular complex detection (MCODE) plugin of the software Cytoscape. (**a**) Key PPI networks for the up-regulated DEPs in the aborted placentas; (**b**) Key PPI networks for the down-regulated DEPs in the aborted placentas

Protein to protein interaction (PPI) networks were established with Cytoscape software for the up- and down-regulated DEPs respectively, to better understand interactions of the candidate functional proteins. The main functional modules of the PPI networks were obtained through filtered by Molecular Complex Detection (MCODE) (Fig. 5).

There are obviously two grouped functional clusters (Fig. 5a), which are mainly responsible for vertebrate embryonic development (Cluster 1) and ubiquitinated protein degradation (Cluster 2), for the up-regulated DEPs in the aborted placentas. These finding suggest that the up-regulated DEPs took parts in protein metabolic disturbance and foetus developmental abnormality.

At the same time, the nine major clusters (Cluster 3 ~ 11) are generated from the PPI networks of the down-regulated DEPs in the aborted placentas (Fig. 5b). Cluster 4, Cluster 9 and Cluster 10 seem to be responsible for protein metabolism because these clusters include proteins (PSMD7, PSMB3, PSMC3, PSMD6, UBLCP1, UBQLN1, UBFD1, AFG3L2, CLPP, PMPCB, PITRM1, GCDH, BCAT1, ASS1, GOT1 and GOT3) that are involved in “proteasomal protein catabolic process” and “proteolysis process”. Cluster 8 and Cluster 9 contain many filtered proteins (ACSL5, ACSL6, PCCB, HADH, HIBADH, CPT2 and ACADM) that participate in “lipid metabolism process”. Cluster 6, Cluster 9 and Cluster 10 comprise several proteins (GNS, PSAP, ETFB, PC, DLAT, FBP1, LAP3, GAPDH and IDH1) that are implicated in “carbohydrate metabolic process”. There are many filtered proteins (LHPP, PPA2, SORD, SOD1, ALDH7A1, and PRDX2) in Cluster 7 and Cluster 10, which take part in “oxidation–reduction process”. In addition, the proteins in Cluster 3, Cluster 5 and Cluster 11 play crucial roles in “cytoskeleton construction”, “RNA transport” and “transcription” respectively. These key PPI networks indicate that the down-regulated DEPs in the aborted placentas aggregate separately, but interact one another in each cluster.

## Discussion

Miscarriage is a major source of reproductive wastage in yak, accounting for massive economic losses to yak industry, which severely restricts yak population reproductive capacity. It is essential to understand the pathological mechanism underlying yak miscarriage, and further to provide some valuable methods to prevent yak miscarriage. The dynamic balance of fetomaternal interaction through placenta plays a crucial role in control of critical biological processes, including embryo implantation, pregnancy maintenance and parturition initiation [22, 23]. A Spontaneous miscarriage will take place when the dynamic balance disorders during gestation period [24].

Although spontaneous miscarriage occurs due to a wide range of causes, alterations in placental function dramatically affect foetal ability to adapt to intrauterine environment [25]. Discovering abnormally expressed proteins in aborted placenta is highly beneficial for enhanced understanding of the pathophysiological mechanism underlying pregnancy loss in yak. Expression patterns of proteins from aborted placentas can be characterized by differential proteomic analysis. It has been reported that iTRAQ proteomics can be applied to screen for protein markers related to early recurrent spontaneous abortion [26, 27] in human. However, little is known about biomarkers associated with spontaneous miscarriage and mechanism underlying spontaneous miscarriage in yak. Understanding of the biochemical pathways affecting the final stage of spontaneous miscarriage process was of paramount value for elucidating the abortive pathogenesis during late pregnancy in yak.

The previous reports show weight-specific rate of protein breakdown is lower in idiopathic recurrent spontaneous miscarriage in the first trimester, and then increases to levels comparable to those in the pregnant counterparts in the third trimester of human pregnancy [28, 29]. The gestation-related changes in protein turnover may be a consequence of greater demands for amino acids imposed on mother by foetus. In the present study, protein and amino acid metabolic pathways were enriched based on the DEPs from the aborted yak placentas. The proteins implicated in protein catabolic processes (“negative regulation of proteasomal protein catabolic process”, “negative regulation of proteolysis involved in cellular protein catabolic process”, “negative regulation of cellular protein catabolic process” and “peptidase activity”) were down-regulated in the aborted placentas (Additional file 7), suggesting that amino acid shortage might have taken place in the yak miscarriages due to the impaired protein catabolism.

Plasma alanine level was significantly lower in the pregnant women for the first 60 h of fasting than those in the nonpregnant counterparts [30]. Lower alanine metabolic level was also demonstrated by KEGG analysis which enriched “alanine, aspartate and glutamate metabolism” pathway for the down-regulated proteins in our study (Fig. 4b), indicating a detriment of alanine metabolic abnormality in placenta to foetus survival. In the pathway, BCAT1 that is a branched-chain amino acid transaminase was down-regulated significantly in the aborted placentas. Diseases associated with BCAT1 include hypervalinemia. HIBADH that plays a critical role in the catabolism of valine [31] also reduced its expression level. Therefore, the decline of BCAT1 and HIBADH might contribute to the abnormal alanine level. Elevated levels of arginine, phenylalanine and tyrosine in serum of women with idiopathic recurrent spontaneous miscarriage were found as compared with those of fertile controls [32, 33]. However, decreased expression of arginine in the aborted yak placentas were discovered by the pathways, “arginine biosynthesis” and “arginine and proline metabolism”, enriched by KEGG analysis based on the down-regulated proteins (Additional file 9). ASS1 that catalyses the penultimate step of the arginine biosynthetic pathway declined in abundance in the aborted yak placentas, which might act as a key factor lead to the decreased arginine level in the pathways. In contrast to the previous studies, the pathway “phenylalanine, tyrosine and tryptophan biosynthesis” was found in the enrichment result of the down-regulated proteins (Additional file 9), implying declined levels of phenylalanine and tyrosine in the aborted yak placentas. These results suggest that metabolic disorders of arginine, phenylalanine and tyrosine might lead to miscarriage whether they are up- or down-regulated.

Homocysteine, an intermediate of methionine metabolism, is implicated to pregnancy-related disorders such as preeclampsia, spontaneous abortion, placental abruption and premature delivery. Homocysteine can be remethylated back to form methionine or metabolized via transsulfuration pathway to generate cystathionine and then to cysteine and α-ketobutyrate. Plasma concentrations of total cysteine and homocysteine in pregnant women are lower during their gestation [28]. High transsulfuration rate in the first trimester can provide cysteine to foetus and result in an obligatory requirement for methionine. Integrated metabolism of methionine provides a sustenance of the one-carbon pool, which is critical for supply of methyl groups required to meet demand of purine synthesis and for the numerous methylation reactions [34]. In the present study, the enriched pathway “cysteine and methionine metabolism” (Fig. 4b) for the down-regulated proteins in aborted placentas indicated the weakened metabolisms of cysteine and methionine. The lower methionine metabolic level demonstrated changes in the one-carbon metabolism, suggesting that the weakened methyl supply derived from abnormal methionine metabolism may result in yak miscarriage.

Occurrence rate of clinically spontaneous abortion for insulin-dependent diabetic women has been determined to be twice as frequent as for general women [35], implying that maternal saccharometabolic status plays a momentous role in pregnancy process. In the present study, AKR1B1 and HKDC1 were down-regulated in the aborted placenta tissue. AKR1B1 is implicated in development of diabetic complications by catalysing reduction of glucose to sorbitol [36]. Reduced HKDC1 expression is also associated with gestational diabetes mellitus [37]. The abnormal downregulation of the two proteins imply that the abortive yaks shared some glucometabolic features with diabetic patients. On the other hand, pregnant women are more sensitive to fasting than non-pregnant females. Plasma glucose concentrations of pregnant women is significantly lower than those of control group at the first stage of fasting [30]. Furthermore, pregnant women tend to suffer hypoglycemia despite a greater gluconeogenesis than non-pregnant control [30].

Abnormal glucose metabolism in human uterus is associated with several pregnancy complications, and also exists in patients with poor fertility [38, 39]. Glucose metabolism is activated for human decidualization [40], and decidualization requires enhanced glycolysis [41, 42]. Disrupting glycolysis impairs decidualization both in vitro and in vivo [43] and impaired decidualization is an essential reason for miscarriages [44]. In line with the results of the previous studies, the abnormal glycometabolism was also discovered in the biological processes enriched by GO analysis in the present study, namely “carbohydrate metabolic process”, “hexose metabolic process” and “monosaccharide metabolic process” (Additional file 7). Likewise, KEGG analysis also enriched many glycometabolism pathways, including “fructose and mannose metabolism”, “glycolysis/gluconeogenesis”, “amino sugar and nucleotide sugar metabolism” and “galactose metabolism” (Fig. 4b). In brief, the glycolysis pathway and the other glycometabolism pathways were perturbed in the aborted yak placentas. These results can be confirmed by the downregulation of many proteins playing crucial roles in the above pathways, including DLAT, FBP1, GAPDH, FH, PC, PGM2 and SORD. The results of our research indicate that abnormal glycometabolism in placenta might induce to yak miscarriage.

Proper growth of human embryo requires adequate lipid supply as signal molecules or nutrient support [45–47]. Sphingolipid metabolic pathway is highly activated in decidua during normal mouse pregnancy. Disturbance in the activated pathway alters levels of key sphingolipid signalling metabolites, causing defects in decidual cells and decidual blood vessels, even early pregnancy losses [48]. The “sphingolipid signalling pathway” was also obtained through KEGG analysis for the up-regulated proteins in the aborted yak placenta (Fig. 4a). The result suggests excessive activation of the “sphingolipid signalling pathway” in placenta might induce abortion either. On the other hand, lack of essential fatty acids, high ratio of C20:4n-6/C20:5n-3, abnormal eicosanoids metabolism and low content of lysophosphatide and diacylglycerol in placenta are potential risk factors for early spontaneous human pregnancy loss [49]. In the present study, the “fatty acid degradation” and “fatty acid biosynthesis” pathways were abnormal in the aborted yak placentas (Additional file 9), suggesting impaired fatty acid metabolism may lead to spontaneous abortion. In the pathways, the representative proteins, CPT2, HADH, ASCL5, ASCL6, ACOX1 and ACADM, that demonstrated markedly lower expression levels in the aborted yak placentas, take parts in fatty acid oxidization and degradation. The disordered expression of these functional proteins was likely to influence normal yak pregnancy. Especially, ACADM defect causes medium-chain acyl-CoA dehydrogenase deficiency, which can result in infantile death [50]. ASAH1 and PLA2G15 can transfer other biomacromolecules to free fatty acid. For example, ASAH1 catalyses degradation of ceramide into sphingosine and free fatty acid [51], and PLA2G15 hydrolyses lysophosphatidylcholine to glycerophosphorylcholine and free fatty acid. The decreased expression levels of the two proteins imply low abundance of free fatty acid in the aborted yak placentas, which might have leaded to the yak miscarriages on account of deficient fatty acid.

Metabolic syndrome is often accompanied by protein, carbohydrate or lipid metabolic disorders. Pathophysiological phenomenon of metabolic syndrome complications are mainly elevated free radicals and common inflammatory stress [52]. Metabolic syndrome is regarded to impair reproduction with increased oxidative stress and burden of inflammation [53]. Oxidative stress induced by increased free radicals contributes to development of pro-oxidative milieu, leads to biomolecules injuries [54], boosts tissue and cell impairment, and eventually induces injury in placentation [55, 56]. The oxidative ingredients are formed through various pathways such as oxidative phosphorylation in mitochondria. In the present study, the “oxidative phosphorylation” pathway is shown in the KEGG analysis for the down-regulated proteins (Fig. 4b), which might have caused redox imbalance detrimental to the yak placenta function.

An imbalance between reactive oxygen species and antioxidants leads to damage to nucleic acids, proteins and lipids [57]. Precocious exposure of embryos to ambient oxygen concentrations often increase rate of congenital malformations and risk of pregnancy termination [58, 59]. Hypoxia can also cause impaired placenta function and foetal growth restriction. Placental oxidative injury generated from ischemia–reperfusion happens in human when hypoxic placental tissues are reoxygenated [60]. Feto-placental unit produces plentiful antioxidants to retain oxidative stress under control. Lack or shortage of antioxidant has been demonstrated to be associated with recurrent pregnancy loss in human [61], suggesting that oxidative stress overwhelms antioxidant defense system. Absence of transsulfuration activity in foetal liver leads to inability of foetus to synthesize cysteine which is a key component of glutathione [62]. Glutathione demand from developing embryo is demonstrated by the data relating oxidant injury to foetal malformations [63]. Identical to the previous researches, the disturbed “glutathione metabolism” pathway was disclosed by our KEGG analysis for the down-regulated proteins in the aborted placentas (Additional file 9), implying that the antioxidant capacity did not keep pace with the increased oxygen tension leading to an increasing oxidative stress on the yak placentas.

Many antioxidative proteins have also been discovered in maternal-foetal interface. Downregulation of FTO in chorionic villi disrupts immune tolerance and angiogenesis at maternal-foetal interface, resulting in increasing oxidative stress that eventually leads to spontaneous abortion [64]. As important antioxidants in biochemical reactions, SOD catalyses conversion of superoxide anions to hydrogen peroxide, and CAT converts the produced hydrogen peroxide to water. The SOD and CAT can be generated to compensate for enhanced levels of reactive oxygen species in placenta. Their decreased levels are found as the previous studies stated in the patients with recurrent pregnancy loss [65, 66]. The reduced FTO, SOD and CAT levels had also been observed in the aborted yak placentas. The declining antioxidant levels may result in the abnormal placentation, syncytiotrophoblast destruction, and subsequently miscarriage during yak gestation. In summary, results of the present study showed the remarkable decreased levels of anti-oxidant elements in the abortive group. This condition may disrupt the balance between oxidative and antioxidative factors and lead to more oxidative damage in yak placenta.

Although the present study contributes to elucidate the pathogenesis of spontaneous miscarriage in yak, it still has some limitations that need to be taken into account. The relatively low number of individual samples per group might have caused inaccurate conclusions if the selected yaks were not representative of the population. Studies with a larger sample size are expected to confirm the findings in the present study. Yak placenta is a set of mixture of cells, containing various cell types. So, the identified DEPs derived from the average expression level difference of all the cells in each placenta sample. It cannot be inferred which cell type contributes to the primary expression alteration. The DEPs were discovered between the aborted and normal placentas by differential proteomics, but it remains to be determined whether these proteins contribute directly to yak miscarriage, or exist merely as biomarkers. The functions of these DEPs in yak placentas remain unclear, particularly of the novel DEPs. For more reliable results, an in-depth study is further required to more accurately understand the functions of DEPs in yak placenta. In addition, although the normal placentas and aborted placentas were expelled at the similar period, the proteomic dynamics might have altered subtly between the different periods.

## Conclusion

Overall, this study filtered 415 DEPs between normal and aborted placentas in yak, which participate directly or indirectly in the non-infectious spontaneous miscarriages. The DEPs of the aborted placentas could be considered as placental protein markers for yak miscarriage. The total DEPs were mainly enriched to lipid, saccharide and protein metabolic process and oxidoreduction pathway, suggesting that metabolic disorders of biological macromolecules and redox imbalance in placenta might cause non-infectious spontaneous miscarriage in yak.

## Methods

### Sample collection and preparation

The study was performed in the Yak breeding and Extending Service Centre in Qinghai Province (Datong country, Qinghai province, China). The yaks with a similar gestation phase (two to three months), free of disease, were selected as potential targets for placenta sample collection. There is not genetic relationship among the female yaks and their breeding males. The candidate yaks were raised in a warm shed pen with regular feeding three times a day (8:00 a.m., 2:00 p.m. and 8:00 p.m.), free access to drinking water. The miscarriage yaks expelling placentas at gestation about month eight were defined as an abortive group. The yaks expelling normal placentas at term were considered as a normal group. The placentas were collected immediately after expelled. Placenta tissues from six yaks, including aborted (*n* = 3) and normal samples (*n* = 3), were collected. These placenta samples were immediately minced into pieces, put into a cryogenic tube, and then frozen in liquid nitrogen as soon as possible.

The yaks were handled in strict accordance with good animal practices by following the Animal Ethics Procedures and Guidelines of the People's Republic of China. The present study was approved by the Animal Administration and Ethics Committee of Lanzhou Institute of Husbandry and Pharmaceutical Sciences of Chinese Academy of Agricultural Sciences (Permit No. SYXK-2019–0032).

### Protein preparation and isobaric tags labelling for relative and absolute quantification

The placenta tissues were ground in liquid nitrogen into cell powder and then disintegrated by lysis buffer (8 M urea in 150 mM Tris–HCl, pH 8.0, 1% Protease Inhibitor Cocktail). The samples were sonicated (15 s, 3 min) three times on ice using a high-intensity ultrasonic processor (Sonics, USA) and centrifugation at 12,000 g at 4 °C for 10 min. The supernatant was precipitated overnight with 5 × volume of chilled acetone and re-suspended in 0.5 M triethylammonium bicarbonate containing 0.5% sodium deoxycholate. The protein concentration was determined with a Bradford kit (Solarbio Co., Beijing, China) according to the manufacturer’s instructions. Subsequently, one hundred micrograms of protein from each sample were mixed with 40 μL dissolution buffer containing 2 μg trypsin and were digested overnight at 37 °C. The digests were then dried by vacuum centrifugation and processed under the manufacturer’s protocols for an 8-plex iTRAQ reagent (AB SCIEX, Foster City, CA, USA). The digested peptides from the three normal placenta samples were labelled with iTRAQ tags 115, 119, 117, and those from the three aborted placenta samples were labelled with iTRAQ tags 118, 116, 121. The labelled samples were then pooled, dried by vacuum centrifugation, and fractionated by SCX chromatography on an AKTA Purifier (GE Healthcare, Fairfield, CT, USA). The elution was monitored at 214 nm, and the fractions were collected every minute for a total of 36 fractions. The fractions were then combined into 20 pools, desalted on standard density Empore SPEC18 Cartridges (Sigma, Santa Clara, CA, USA) with inner diameter 7 mm and volume 3 mL, concentrated by vacuum centrifugation, and reconstituted in 40 mL 0.1% formic acid.

### Liquid chromatography-mass spectrometry analysis

LC–MS analysis was performed on a Q Exactive mass spectrometer (Thermo Fisher Scientific, San Jose, CA, USA) coupled to an Easy nLC (Proxeon Biosystems, now Thermo Fisher Scientific). Mass spectra were collected (350–1,500 m/z) at high resolution (> 60,000) for 30 s per spectrum. The automatic gain control target was set to 3 × 10^6^, and maximum injection time to 10 ms; dynamic exclusion duration was 40.0 s. Survey scans were acquired with a resolution of 70,000 at 200 m/z, the resolution for HCD spectra was set to 17,500 at 200 m/z, and the isolation width was 2 m/z. The normalized collision energy was 30 eV, and the underfill ratio which specifies the minimum percentage of the target value likely to be reached at maximum fill time was defined as 0.1%. The instrument was run with peptide recognition mode enabled. LC–MS were performed by BGI Biological Technology Co.Ltd. (Beijing, China).

### Protein identification and quantitation

The raw LC–MS data were converted into MGF format by Proteome Discoverer (Thermo Fisher Scientific Inc. San Jose, CA, USA), and then matched to peptide sequences via database search using Mascot (version 2.3.02) [67]. ProteinPilot 4.5 software (ABSciex) was applied to identify and quantify proteins based on the recorded MS/MS spectra against yak proteomic data downloaded from the Uniprot database. Carbamidomethylation on cysteines was set as fixed modification, while methionine oxidation, N-acetylation and phosphorylation were considered as variable modification. Cysteine alkylation modification was defined as methylmethanethiosulfate. The tag type was set to iTRAQ 8 plex. Trypsin was selected as digestion enzyme. A mass tolerance of 20 ppm for peptide precursors and 0.1 Da for fragmented ions was set, with allowance for one missed cleavage in the trypsin digests. The common target-decoy search approach was adopted to evaluate FDRs for peptide identification based on database searching against the reversed protein sequences. The peptide for quantification was automatically selected by Pro Group algorithm to calculate the reporter peak area, error factor and *p*-value.

### Differentially expressed proteins screening

For protein quantitation, a minimum of one unique peptide was required. Fold change of each protein was calculated by comparing summed ion intensities of unique peptide/peptides for each protein from the two sample groups. Statistic significant differences between the two groups were determined with the Student’s t-test in R software environment (version 4.0.3). A fold change above 1.2 and a *p*-value below 0.05 between the two groups were adopted as thresholds to obtain DEPs.

### Expression pattern analyses for the identified proteins

The R package “ggplot2” was used to generate a box plot to display the protein expression patterns in the normal and aborted yak placentas. The R package “FactoMineR” was applied to conduct Hierarchical agglomerative clustering and the R package “factoextra” was used to generate PCA biplot based on the expression matrix of all the identified proteins. Hierarchical clustering and heatmap visualization for all the identified proteins were performed by the R package “stats” according to hierarchical clustering on the basis of the expression matrix. The R packages “ggpubr” and “ggthemes” were applied to construct volcano plot for all the identified proteins. A heatmap of the DEPs was plotted by the R package “stats” as above described.

### Gene ontology and Kyoto encyclopaedia of genes and genomes analyses for the differentially expressed proteins

In order to better understand the biological functions of DEPs and pathways they are involved in, GO and KEGG pathway enrichment analyses [68–70] were carried out based on the up- and down-regulated DEPs in the aborted yak placentas. GO and KEGG terms were enriched using the R package “clusterProfiler”, “GO.db” and “org.Bt.eg.db” to identify significant BPs, CCs, MFs, and KEGG pathways. The bubble diagrams were then created by the R package “ggplot2” to exhibit significant enrichment for the GO and KEGG terms of the up- and down-regulated DEPs respectively.

### Protein to protein interaction analysis for the targeting differential expressed proteins

Protein to protein interaction analysis was performed to identify relationships among hub DEPs, to find functional proteins with key roles in spontaneous miscarriage and to understand potential pathological mechanism underlying yak miscarriage. The PPI networks were constructed separately for the up- and down-regulated DEPs in aborted placentas by STRING plugin [71] of Cytoscape software (version 3.8.2) [72]. The functional modules of the PPI network were subsequently sifted by MCODE plugin of Cytoscape.

## Supplementary Information

**Additional file 1.** Annotated peptides and their mass spectrometry information.

**Additional file 2.** Identified proteins and their annotation information.

**Additional file 3.** Trusted proteins and their annotation information.

**Additional file 4.** Up-regulated differentially expressed proteins and their annotation information.

**Additional file 5.** Down-regulated differentially expressed proteins and their annotation information.

**Additional file 6.** Gene ontology enrichment results for the up-regulated differentially expressed proteins.

**Additional file 7.** Gene ontology enrichment results for the down-regulated differentially expressed proteins.

**Additional file 8.** Kyoto encyclopaedia of genes and genomes enrichment results for the up-regulated differentially expressed proteins.

**Additional file 9.** Kyoto encyclopaedia of genes and genomes enrichment results for the down-regulated differentially expressed proteins. 

## Data Availability

The mass spectrometry proteomics data generated during the current study are available in the ProteomeXchange Consortium via the iProX partner repository, [http://proteomecentral.proteomexchange.org/cgi/GetDataset?ID=PXD025428]. Other raw datasets may also be requested from the corresponding author provided that all ethical requirements have been met.

## Abbreviations

iTRAQ: Isobaric tags for relative and absolute quantification
DEP: Differentially expressed protein
*C. abortus*: *Chlamydia abortus*
PCA: Principal component analysis
GO: Gene ontology
KEGG: Kyoto encyclopaedia of genes and genomes
PPI: Protein–protein interaction
MCODE: Molecular complex detection
LC–MS: Liquid chromatography-mass spectrometry
FDR: False discovery rate
BP: Biological process
CC: Cellular component
MF: Molecular function
